# The emerging role of oncolytic virotherapy in genitourinary malignancies

**DOI:** 10.3389/fcell.2026.1830852

**Published:** 2026-06-15

**Authors:** Juliana Baboghlian, Jasmine Vohra, Lucas Assoni, Marina S. Folguieri, Guilherme Z. Rocha, Jaime Henrique Amorim, Shahrokh F. Shariat, Leonardo O. Reis

**Affiliations:** 1 UroScience, School of Medical Sciences, University of Campinas (UNICAMP), São Paulo, Brazil; 2 INCT-UroGen, National Institute of Science, Technology and Innovation in Genitourinary Cancer, São Paulo, Brazil; 3 Department of Biomedical Engineering, Vidyalankar Institute of Technology, Mumbai, Maharashtra, India; 4 Western Bahia Virology Institute, Center of Biological Sciences and Health, Federal University of Western Bahia, Barreiras, Bahia, Brazil; 5 Department of Urology, Comprehensive Cancer Center, Medical University of Vienna, Vienna, Austria; 6 Institute for Urology and Reproductive Health, I.M. Sechenov First Moscow State Medical University, Moscow, Russia; 7 Department of Urology, Lazarski University, Warsaw, Poland; 8 Department of Urology, Weill Cornell Medical College, New York, NY, United States; 9 Department of Urology, University of Texas Southwestern, Dallas, TX, United States; 10 College of Medical Health Technologies, Al-Turath University, Baghdad, Iraq; 11 Hourani Center for Applied Scientific Research, Al-Ahliyya Amman University, Amman, Jordan; 12 Karl Landsteiner Institute of Urology and Andrology, Vienna, Austria; 13 ImmunOncology, Pontifical Catholic University of Campinas (PUC-Campinas), São Paulo, Brazil

**Keywords:** bladder cancer, genitourinary cancers, immunogenic cell death, immuno-oncology, oncolytic viruses, prostate cancer, renal cell carcinoma, virotherapy

## Abstract

Genitourinary cancers represent a significant global health burden, accounting for millions of new cases annually and high mortality, particularly in advanced disease. Conventional therapies such as surgery, chemotherapy, radiotherapy, and immune checkpoint inhibitors have improved outcomes but are often limited by resistance, tumor immune evasion, and treatment-related toxicities. Oncolytic viruses, with their dual capacity to directly lyse tumor cells and stimulate systemic anti-tumor immunity, have emerged as a promising therapeutic platform in genitourinary oncology. This review provides a comprehensive analysis of the mechanistic underpinnings of oncolytic viruses’ therapy, encompassing direct oncolysis, immunogenic cell death, modulation of the tumor microenvironment, and genetic engineering strategies. We highlight key viral platforms, landmark clinical advances, and future perspectives in personalized therapies, CRISPR-based genetic engineering, and biomarker-driven clinical translation. Taken together, the integration of oncolytic viruses into genitourinary cancer management represents a rapidly maturing field, with the potential to transform current therapeutic paradigms through tumor-selective viro-immunotherapy.

## 1 Introduction

Genitourinary (GU) malignancies, including prostate cancer (PC), bladder cancer (BC), and renal cell carcinoma (RCC), represent a significant burden on global health, accounting for a substantial proportion of cancer-related morbidity and mortality in men worldwide. Many GU tumors display primary or acquired resistance to immunotherapy and a high capacity to resist chemotherapeutic agents, largely due to the immunosuppressive effects of the tumor microenvironment (TME) and the adaptation and evasion of tumor cells to drugs (Zhang and Rabkin, 2021; Sharma et al., 2021; Wang Z. et al., 2025; Rinaldi et al., 2025; Hasan et al., 2025; Dyrskjøt et al., 2023; Lopez-Beltran et al., 2024; Al Hussein Al Awamlh and Chang, 2023). This persistent therapeutic gap has stimulated growing interest in immunotherapy with oncolytic viruses, a rapidly evolving platform that exerts a dual mechanism of action, direct lysis of tumor cells and robust activation of antitumor immune responses, making it particularly suitable for reprogramming the immunosuppressive TME in GU cancers and addressing the current limitations of standard treatment (Zhang and Rabkin, 2021; Kennedy et al., 2020).

PC remains the most diagnosed neoplasm among men in many regions and remains among the leading causes of cancer-related death worldwide. According to the International Agency for Research on Cancer (GLOBOCAN), an estimated 1.46 million new cases and 397,743 deaths related to PC were tallied in the last published report, ranking fourth and eighth in overall rates of new cases and mortality (Bray et al., 2024). Global inequality in access to curative and preventive medicine leads to highly variable PC incidence rates, with the highest rates observed in countries with better economic conditions, where diagnosis occurs earlier, and treatment is more effective (Halaseh et al., 2023).

According to the World Bank Atlas classification, countries can be grouped by gross national income *per capita* into high-income, upper-middle-income, lower-middle-income, and low-income categories (Metreau et al., 2024). The high mortality rates observed locally in Lower-middle-income and Low-Income countries are extremely concerning, as they do not correspond to the incidence rates, often due to late diagnosis and scarcer treatment resources for a considerable portion of their population (Halaseh et al., 2023).

Prostate-specific antigen (PSA) is widely used as a noninvasive screening test for PC detection, especially for indolent disease. Another important marker is Prostate-specific membrane antigen (PSMA), a transmembrane glycoprotein widely used as a biomarker for PC due to its stage-dependent overexpression in cancer cells. This expression profile enables the evaluation of tumor progression and disease evolution (Altunay et al., 2025; Wang H. et al., 2024). Although PSMA is also expressed in other tissues, its role as a biomarker has been extensively validated in PC (Avilez et al., 2026). Nevertheless, certain subsets of PC patients, particularly those with castration-resistant or neuroendocrine disease, may exhibit lower PSMA expression levels (Bakht and Beltran, 2025). The conjugation of PSMA-targeting agents with radioisotopes for positron emission tomography computed tomography (PET/CT) has substantially improved the detection and monitoring of tumor progression and metastatic lesions. Compared with conventional imaging modalities, such as magnetic resonance imaging (MRI), PSMA PET/CT demonstrates higher sensitivity, enabling more accurate lesion detection, improved tumor staging, and better-informed clinical decision-making (Wang H. et al., 2024; Jochumsen and Bouchelouche, 2024).

Given these properties, several PSMA-targeted radionuclide therapies have also been developed and advanced to clinical trials, with one agent (Lu^177^–PSMA-617) receiving approval in 2022. Therapeutic strategies have included the use of PSMA-radionuclide conjugates as monotherapies or in combination regimens. Additional approaches include anti-PSMA radioimmunotherapy and radioguided surgery (Wang H. et al., 2024; Jang et al., 2023).

Despite advances in therapies, including androgen deprivation therapy, second-generation antiandrogens (e.g., abiraterone, enzalutamide), chemotherapy, radiopharmaceuticals, and immunotherapies that are currently making significant progress in melanoma, lung cancer, and urothelial carcinoma, the progression to castration-resistant prostate cancer (CRPC) remains common and generally incurable. Furthermore, PCs have insufficient infiltration of immune cells to elicit robust responses due to the immune checkpoint blockade. Techniques are being developed to reprogram the TME to overcome these challenges; however, this resistance has emerged as a major obstacle to long-term control in metastatic settings (Kwo et al., 2025).

BC is a prominent GU malignancy, which ranks as the ninth most common cancer globally, with 614,298 cases and 220,596 deaths in 2022 (Bray et al., 2024). 90% of BC are classified, according to cellular origin, as urothelial cell carcinoma (Lopez-Beltran et al., 2024). Regarding staging, it can be classified as non-muscle-invasive bladder cancer (NMIBC), characterized when the neoplasm is located in the mucosa and submucosa of the bladder tissue, and as muscle-invasive bladder cancer (MIBC), showing a greater infiltrative capacity, reaching the muscular and other deeper layers, while also possessing a more expressive number of gene mutations, which are associated with the metastatic phenotype (Hasan et al., 2025; Dyrskjøt et al., 2023; Lopez-Beltran et al., 2024; Al Hussein Al Awamlh and Chang, 2023).

Approximately 70%-75% of BC are NMIBC type tumors, which, in general, are treated with transurethral resection followed by intravesical *Bacillus* Calmette–Guérin (BCG) therapy. MIBC-type tumors require more intensive treatment with neoadjuvant or adjuvant systemic chemotherapy along with radical cystectomy, which may also be associated with radiotherapy (Lopez-Beltran et al., 2024). Common comorbidities of MIBC, such as renal failure, make cisplatin-based chemotherapeutic agents ineligible. Similarly, in NMIBC tumors, some patients are refractory to BCG treatment; in these cases, pembrolizumab, an anti-programmed cell death protein 1 monoclonal antibody (anti-PD-1 mAb), is an option (Hasan et al., 2025; Lopez-Beltran et al., 2024). This also highlights the need for more therapeutic alternatives for BC, especially bladder-sparing approaches.

Neoplasms located in the kidneys rank third in incidence of GU cancers, accounting for 434,840 cases and 155,953 deaths according to GLOBOCAN (Bray et al., 2024). RCC originates in the epithelium lining of the proximal renal tubules, is extremely aggressive, and is often diagnosed incidentally during imaging exams. The most frequently found histological type is clear cell renal carcinoma (ccRCC) (80%), which is generally associated with a poor prognosis (Sharma et al., 2021; Rinaldi et al., 2025). Even in cases where the diagnosis is made at localized or locally advanced stages and curative surgical resection is performed, the incidence of metastases and recurrence is approximately 30% (Sharma et al., 2021; Wang Z. et al., 2025). All types of RCCs are resistant to conventional treatments such as chemotherapy and radiotherapy (Sharma et al., 2021). For this reason, targeted therapies, such as molecular therapies, e.g., vascular endothelial growth factor tyrosine kinase inhibitors - VEGF-TKIs or immune checkpoint inhibitors–ICIs (Sharma et al., 2021), are being used and developed, aiming to increase the survival time of patients. However, even these types of therapies also present resistance, making treatment still challenging as PC (Wang Z. et al., 2025).

## 2 Oncolytic virus therapy

The concept of using viruses to treat cancer dates back more than a century, with sporadic reports of tumor regression during viral infections. However, only with the evolution of virology, tumor immunology, and genetic engineering has this idea evolved into a clinically viable strategy (Zhou et al., 2025), as oncolytic virus (OV) immunotherapy uses native or genetically modified viruses for cancer treatment (Kaufman et al., 2015). Despite the ability of viruses to enter both normal and cancerous cells, the alterations found in the latter make them more prone to viral multiplication due to changes triggered by stress, type of cell signaling and homeostasis mechanisms, as well as failures in antiviral defenses, such as, the protein kinase R, which plays a role in the elimination of intracellular viruses, may be absent or altered, promoting greater viral replication (Zhou et al., 2025).

The most widely used viral platforms in oncolytic virotherapy include adenoviruses, herpes simplex virus type 1 (HSV-1), reoviruses, and poxviruses, which are generally used in combination with other interventions to improve the patient’s chances of effective treatment (Zhou et al., 2025). One of the most significant milestones in the use of OVs in immunotherapy was the approval of Talimogene Laherparepvec (T-VEC) in 2015 in the United States, a herpes simplex virus type 1 (HSV-1) genetically modified to express granulocyte-macrophage colony-stimulating factor (GM-CSF), for the treatment of unresectable melanoma. This represented a paradigm shift, as OVs were no longer seen merely as oncolytic agents, but as immunostimulatory biological agents capable of initiating tumor-specific systemic immunity (Kaufman et al., 2022; Yin and Wang, 2024).

Recent advances have further enhanced OV precision and efficacy, including synthetic biology approaches that enable multi-gene payload engineering and modular immune-modulatory strategies. Armed viruses can deliver localized immune modulators directly into the tumor bed. Additionally, combination strategies with ICIs, radiation, or chemotherapy are being explored to enhance efficacy in tumors that are otherwise resistant. These advances are particularly relevant to GU cancers, which often exhibit immune exclusion or immune suppression, respond poorly to checkpoint blockade alone, and allow direct tumor access for local OV administration. Thus, OVs offer a unique therapeutic modality, as they can reprogram immune-inert into immune-reactive tumors, potentially unlocking responses to subsequent or concurrent immunotherapy (Hu et al., 2022; Kumar et al., 2021).

### 2.1 Oncolytic viruses mechanisms of action

OVs represent a distinct class of immunotherapeutic agents that not only mediate direct tumor cell destruction through replication-dependent cytolysis but also initiate robust immune responses by enhancing tumor antigen exposure and reprogramming the TME (Nguyen et al., 2022). Their multimodal mechanisms, including direct oncolysis, induction of immunogenic cell death (ICD), recruitment of immune effector cells, and amenability to genetic engineering, have generated substantial interest in their capacity to convert immunologically “cold” tumors into immunologically “hot” tumors, as evidenced by encouraging results reported in both monotherapy and combination treatment settings (Harrington et al., 2019).

**FIGURE 1:**
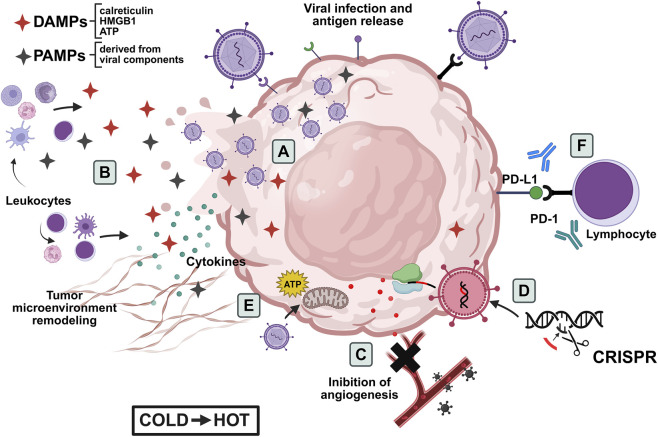
Reprogramming the Tumor Immune Microenvironment Through Oncolytic Virotherapy. The use of oncolytic viruses can modulate the tumor microenvironment, serving as an important tool in cancer treatment and often aiming to transform the TME from “cold” to “hot”. These mechanisms demonstrate this effect [A] Oncolysis through viral multiplication and, consequently, the rupture of the cancerous cell. [B] After oncolysis, DAMPs and viral PAMPs are released, triggering chemotaxis of effector and inflammatory cells, cytokine release, complement system activation, and other immune-activating pathways, also contributing to the remodeling of the tumor microenvironment [C] Some viruses have a natural tropism for the vascular endothelium, causing inflammation and impairing angiogenesis; however, viral gene manipulation can also be used for this purpose. [D] Using the CRISPR technique, it is possible to generate “smart OVs” that preferentially infect tumors with specific molecular signatures or produce therapeutic proteins to aid cancer treatment [E] The metabolic demands of tumor cells may favor viral replication, contributing to selective oncolysis. It is also possible to divert metabolic pathways to increase the efficiency of drugs or make the TME more conducive to fighting cancer through techniques such as CRISPR. [F] Combining oncolytic viruses with immune checkpoint inhibitors (such as anti-PD-1 and anti-PD-L1 antibodies) prevents immune response attenuation.

Figure 1 shows the anti-tumoral mechanisms explored by OVs, which can promote tumor cell elimination through multiple interconnected mechanisms. First, the selective replication within tumor cells of most oncolytic viruses, followed by cellular lysis and viral propagation to adjacent malignant cells, is shown in item A of Figure 1. This selectivity arises from several tumor-specific vulnerabilities, including dysregulated oncogenic signaling pathways, defective antiviral interferon responses, impaired apoptosis, altered gene promoter activity, and defects in DNA damage response (DDR) pathways, among other factors (Zhang and Rabkin, 2021; Alwithenani et al., 2025). Importantly, many tumors exhibit deficiencies in antiviral defense systems that facilitate viral replication while simultaneously increasing susceptibility to immune-mediated elimination (Fayyad-Kazan et al., 2026).

Second, as shown in item B of Figure 1, OVs activate the host immune system by enhancing antigen presentation and stimulating innate immune sensing pathways. Viral infection facilitates the presentation of viral antigens and the release of tumor-associated antigens, thereby stimulating immune recognition and enabling a tumor-specific antitumor response. In this context, cytosolic nucleic acid sensors, particularly cyclic GMP–AMP synthase (cGAS) and stimulator of interferon genes (STING), play a central role in detecting viral or damaged DNA released during OV replication and tumor cell lysis (Wang Q. et al., 2025). Activation of the cGAS-STING pathway promotes phosphorylation of TBK1 and IRF3, leading to type I interferon production and the release of inflammatory cytokines, which enhance dendritic cell maturation and CD8^+^ T Cell priming (Wang Q. et al., 2025; Li et al., 2026). Similarly, RNA-sensing pathways mediated by retinoic acid-inducible gene I (RIG-I) and melanoma differentiation-associated protein 5 (MDA5) recognize viral RNA intermediates generated during replication, further amplifying antiviral and antitumor immune responses (Sanchez David et al., 2016; Dias Junior et al., 2019; Wu et al., 2017). These innate immune signaling pathways are increasingly recognized as critical mediators of OV-induced conversion of immunologically “cold” tumors into inflamed tumors enriched with cytotoxic immune infiltrates.

The interaction between OV infection and signaling also represents an important mechanistic component of OV therapy. DDR pathways involving ATM, ATR, and DNA-dependent protein kinase (DNA-PK) regulate cellular responses to genomic instability and influence both viral replication and immune activation (Yan et al., 2026). Viral infection and OV-mediated cytotoxicity can induce replication stress, DNA double-strand breaks, and micronuclei formation, thereby promoting cytosolic DNA accumulation and activation of cGAS-STING signaling. Conversely, several viruses actively manipulate DDR machinery to optimize replication, while tumors harboring DDR defects frequently display increased permissiveness to OV infection. This bidirectional relationship between DDR and innate immunity has emerged as a major determinant of OV therapeutic efficacy (Marcut et al., 2025).

In this regard, the review by Pateras et al. (Pateras et al., 2015) provides important mechanistic insights into the crosstalk between DDR signaling and immune activation. The authors demonstrate that DNA damage acts not only as a cytotoxic insult but also as an immunological danger signal capable of initiating inflammatory and immune responses through activation of innate sensing pathways. Furthermore, the review highlights how both DNA and RNA viruses interact with DDR components to regulate viral propagation, inflammatory signaling, and cellular immunogenicity. These findings support the concept that OV-mediated oncolysis and radiation-induced DNA damage converge on shared DDR-immune pathways that may synergistically enhance antitumor immunity (Chen et al., 2022).

Third, some engineered OVs can inhibit tumor angiogenesis either through direct effects on the vascular endothelium or *via* genetic modifications that alter endothelial transcription or transduction, resulting in reduced production of angiogenic factors or the expression of antiangiogenic mediators, as represented in item C of Figure 1 (Kennedy et al., 2020; Zhou et al., 2025; Yin and Wang, 2024; Toro Bejarano and Merchan, 2015).

These interactions have stimulated interest in combining OVs with metabolic interventions, such as inhibition of glycolysis through silencing of glycolytic genes (item E, Figure 1), or with immune checkpoint blockade targeting Programmed Cell Death Protein 1/Programmed Cell Death Ligand 1 (PD-1/PD-L1), as illustrated in item F of Figure 1 (Kennedy et al., 2020). Emerging evidence suggests that metabolic modulation may enhance viral replication, reduce immunosuppression within the TME, and improve responsiveness to immunotherapy.

From a translational perspective, increasing attention has focused on combining OVs with radiotherapy due to their complementary immunological and DDR-mediated effects. Ionizing radiation induces DNA damage, replication stress, micronuclei formation, and release of damage-associated molecular patterns (DAMPs), all of which contribute to activation of innate immune pathways, particularly cGAS-STING signaling. Radiation additionally promotes immunogenic cell death characterized by calreticulin exposure, ATP secretion, HMGB1 release, enhanced antigen presentation, and increased chemokine production, thereby facilitating immune cell recruitment into the TME (Png et al., 2025; Zhu et al., 2021; Yadollahvandmiandoab et al., 2022).

Importantly, radiation-induced modulation of the TME may improve OV spread and therapeutic efficacy by enhancing vascular permeability, stimulating inflammatory cytokine production, and increasing tumor immunogenicity (Martinez-Zubiaurre et al., 2018). Simultaneously, OV infection can amplify radiation-induced immune activation by increasing type I interferon signaling and recruitment of cytotoxic lymphocytes. The convergence of OV replication, DDR activation, and innate immune sensing pathways therefore provides a strong biological rationale for OV-radiotherapy combinations (Chen et al., 2026). Preclinical and emerging clinical evidence suggests that these combinations may overcome resistance mechanisms within poorly immunogenic tumors and potentiate systemic antitumor immune responses, including abscopal effects.

### 2.2 The immune landscape of genitourinary cancer

Immunological components have significant prognostic value in cancer and play a critical role in monitoring therapeutic efficacy. Increased infiltration of immune cells, including CD8^+^ T Cells, activated memory CD4^+^ T Cells, and T helper cells, has been associated with improved patient survival outcomes. Cancer-associated fibroblasts (CAFs) are a predominant cellular component of the tumor stroma, and their increased abundance has been correlated with enhanced invasiveness in bladder cancer. Activated fibroblasts can induce epithelial–mesenchymal transition in tumor cells and are also involved in tumor angiogenesis (Yang et al., 2024; Novysedlak et al., 2024). Fibroblasts promote tumor desmoplasia, which limits treatment efficacy. Tumor-associated fibroblasts frequently overexpress fibroblast-activating protein, thereby promoting the formation of a dense collagen-rich extracellular matrix that functions as a physical barrier and impairs immune cell infiltration into the tumor microenvironment (Xiao et al., 2023).

Myeloid-derived suppressor cells (MDSCs) are involved in the regulation of excessive inflammatory responses; however, they also contribute to immune evasion, tumor proliferation, invasion, and metastasis. Tumor-infiltrating lymphocytes (TILs) reflect the host immune response against neoplastic cells and may induce apoptosis through cytokine secretion or death ligand-mediated pathways. Moreover, lymphocytic infiltration has been associated with the immunophenotypic characterization of muscle-invasive bladder cancer and with patient survival. Macrophages secrete several cytokines and growth factors, including TNF, TGF-β, IL-6, and IL-12, and are commonly classified into distinct functional subtypes. Among the cytokines involved in the immune response against GU cancers, interferon expression is essential for activating immune pathways. Gamma interferon (IFNγ) is noteworthy for its central role in host inflammatory responses and its potential as a predictor of therapeutic success. In BC, the predictive value of IFNγ expression is controversial. Some studies have reported that patients with high IFNγ expression exhibit unfavorable immune-related correlates, including increased T- and NK-cell recruitment, altered responses to immunotherapy, enhanced disease progression, and reduced survival, despite decreased expression of immune checkpoint molecules (Deng et al., 2023; Wang J. et al., 2022). Conversely, other predictive models have suggested the opposite association, indicating that elevated IFNγ levels may confer clinical benefit in both NMIBC and MIBC patients (Gillezeau et al., 2022; Baker et al., 2022). Response to type I interferons may also serve as indicators of resistance mechanisms to therapies targeting IFN-based responses, such as Nadofaragene Firadenovec (Green et al., 2021).

In RC, a similar profile has been observed, as the IFNγ response gene expression signature also serves as a predictive marker for survival, immune infiltration, drug sensitivity, and response to ICIs (Liu et al., 2021). In RCC, myeloid-derived IFNγ signaling has been shown to inhibit T-cell-mediated tumor cell killing and promote resistance to ICIs (Bi K. et al., 2025). Other members of the interferon family have also been investigated in the context of BC. Type III interferon (IFNλ), for instance, has been associated with enhanced immune cell activity and improved ICI efficacy by mediating antitumor responses through T Cells and macrophages, highlighting its potential as a therapeutic candidate (Wang B. et al., 2024).

IFNγ treatment in PC has been shown to induce expression of MHC-I and PD-L1, while downregulating E-cadherin, thereby enhancing sensitivity to Camptothecin in metastatic CRPC. Furthermore, *in vivo* models demonstrated that the combination of IFNγ and paclitaxel inhibited metastatic progression (Korentzelos et al., 2022). Similarly, exosomes expressing beta interferon (IFN-β) suppressed PC cell proliferation, reduced tumor size, and increased apoptosis (56). In the context of predictive therapeutic models, increased IFN-β responses following TLR1 and TLR2 activation have also been associated with improved outcomes in metastatic CRPC patients treated with Sipuleucel-T (Brown et al., 2024). M1 macrophages promote acute inflammatory responses, whereas M2 macrophages are associated with chronic inflammation and immunosuppressive mechanisms that may facilitate tumor progression. Regulatory T Cells (Tregs) are recruited by M2 macrophages and secrete granzymes and cytokines capable of suppressing T Cell activation and proliferation. Dendritic cells are essential antigen-presenting cells that actively contribute to antitumor immune responses; consequently, their dysfunction may promote immune evasion by tumor cells (Yang et al., 2024; Novysedlak et al., 2024; Wang et al., 2021).

Immunogenic cell death is a process that relies on the release of endogenous adjuvants to stimulate immune responses against cancer cells, those being the release or surface exposure of key damage-associated molecular patterns (DAMPs), including calreticulin, high-mobility group box 1 (HMGB1), and adenosine triphosphate (ATP). These molecules engage pattern recognition receptors on immune cells, thereby initiating immune activation. In antigen-presenting cells, such as dendritic cells, this process enhances cell recruitment, antigen processing, and its presentation (Yadollahvandmiandoab et al., 2022). Several OVs have been shown to induce the hallmarks of ICD, including DAMP expression and the release of pathogen-associated molecular patterns (PAMPs) derived from viral components, leading to cytokine secretion and subsequent modulation of immune responses (Workenhe and Mossman, 2014; Galluzzi et al., 2017; Pascoal et al., 2025) (item 2, Figure 1). One such example is the telomerase promoter–driven adenovirus OBP-702, engineered to overexpress wild-type p53. In colon and pancreatic cancer models, OBP-702 induced potent ICD by releasing ATP and high-mobility group box 1, accompanied by robust CD8^+^ T Cell infiltration and significant inhibition of tumor growth. In the pancreatic cancer model, combination therapy with OBP-702 enhanced the efficacy of anti-PD-1 blockade (Araki et al., 2022; Yamada et al., 2025).

One of the most clinically relevant effects of oncolytic viruses is their ability to remodel the TME, promoting increased infiltration of inflammatory cells. As highlighted in a recent review by Kong and colleagues, OVs can mediate this transformation by promoting T Cell priming and infiltration within the TME, including by disrupting extracellular matrix–associated barriers that otherwise limit immune cell access (Kong et al., 2025).

Additionally, by enhancing antigen visibility and inflammatory signaling, OVs may improve responses to ICIs, particularly in settings where ICI monotherapy has previously failed. In a phase Ib clinical trial, T-VEC administered in combination with Pembrolizumab demonstrated synergistic antitumor effects. Intratumoral administration of T-VEC in patients with advanced melanoma (n = 21) enhanced the efficacy of anti-PD-1 therapy by modulating the immune ecosystem within the TME (Chesney et al., 2023).

This immunological cascade is critical for overcoming tolerance and the characteristic immunosuppression of GU cancers, particularly prostate tumors, which frequently fail to elicit effective T Cell responses. Accordingly, TME modulation by OVs is not merely a minor benefit but may be a key for unlocking the full therapeutic potential of immunotherapy in GU malignancies (Kwo et al., 2025).

### 2.3 Metabolic reprogramming of tumor cells and their microenvironment

Recent studies show that metabolic alterations in the TME may offer promising avenues for innovation and the enhancement of cancer therapies (Kennedy et al., 2020; Assoni et al., 2026). The subsequent modifications in the tumor cell, such as increased metabolism to obtain energy and substrates, and modulation of the extracellular environment to make it more immunotolerant, are attractive to most pathogens, such as viruses, in addition to the expression of oncogenes or environmental stress, which can activate cellular signaling for the metabolism of glucose, lipids, and proteins. Viral infection also requires a greater energy supply, as in tumor cells, to produce virions and can thus divert the tumor cell’s metabolic pathways to meet its needs. (Kennedy et al., 2020; Wang and Wei, 2022). Examples of metabolic pathway disruptions caused by viruses include the Kaposi’s sarcoma-associated herpesvirus, which induces cellular transformation towards anabolic proliferation, as well as the dysregulation of sensors related to glucose metabolism, thus ensuring the survival of tumor cells and viral replication (Li and Gao, 2021), in another example a study using adenovirus, Ad5 demonstrated that metabolic reprogramming in hepatocellular carcinomas, inducing the production of apolipoprotein-1, can improve the antitumor response by activating CD8^+^ T Cells and stimulating the release of cholesterol from the tumor cell, increasing the expression of immune checkpoint genes and impairment of antitumor activity (Xu et al., 2025).

Genetic engineering is also emerging as an important tool, as viral genes can be manipulated to interfere with the metabolism of cancerous cells. This topic will be discussed in more detail below, adding another important strategy to strengthen antitumor therapies (Kennedy et al., 2020; Wang L. et al., 2022).

### 2.4 General characteristics of strategic oncoviruses

#### 2.4.1 Adenoviruses

As a result of the characteristics of adenoviruses, which combine selective tumor promoters, prodrug-activated cytotoxicity, and immunostimulatory payloads, they become versatile and potent platforms for the treatment of GU cancers (Uchio et al., 2022; Peter and Kühnel, 2020). Adenoviruses are not integrative viruses; they have double-stranded DNA ranging from 30 to 38 kb, are non-enveloped, and are approximately 90 nm in size. Their characteristics, such as the DNA capsid components, enable them to trigger a strong immunogenic response, leading to the release of large amounts of cytokines and chemokines. They can infect various types of epithelial cells. They can be easily genetically altered, potentially adding immune-stimulation genes, controllers of viral replication, tumor lysis amplifiers, extracellular matrix modifiers, T Cell engagers, and deactivators of genes that encode proteins that inhibit premature cell apoptosis (E1), among other strategic targets (Peter and Kühnel, 2020).

When using a recombinant adenovirus encoding the *CXCL10* gene, increased *CXCL10* expression in a murine model of colon carcinoma enhanced the effectiveness of anti-PD-L1 antibody treatment. In this approach, the adenovirus could carry the gene of interest, thereby enhancing the effectiveness of the anti-tumor treatment (Li X. et al., 2022).

#### 2.4.2 Herpes Simplex Viruses

Herpes Simple Viruses (HSV) - based oncolytic viruses, including T-VEC and RP1 (Vusolimogene Orparepvec), stand out for their large genome size (∼150 kb) and neurotropic lytic capacity (Bello-Morales et al., 2021; Zheng et al., 2025). T-VEC is the first FDA-approved OV (melanoma) and combines ICP34.5 deletion (a virus virulence factor associated with neurotoxicity) with GM-CSF expression (an enhancer of the anti-tumor response). Although it has not yet been extensively tested in GU cancers, its success in melanoma suggests potential in PC, particularly if administered intratumorally or intraprostatically to enhance its effect (Zheng et al., 2025).

RP1 is a next-generation immunoncolytic therapy derived from HSV type 1 that expresses GM-CSF and the GALV-GP-R glycoprotein, which has the function of increasing tumor lysis, ICD, and potentiating the systemic antitumor effect (Wong et al., 2025).

#### 2.4.3 Poxviruses

Poxviruses (PXVs) are considered the largest viruses capable of infecting humans, measuring 200-400 nm in diameter. They are enveloped viruses with a double-stranded DNA genome (130–290 kb) and more than 200 open reading frames. PXVs do not depend on the host cell to produce their replication-related essential proteins, nor non-essential proteins associated with virulence. Although they trigger innate immune activation, they can also deploy immune evasion mechanisms (Saghazadeh and Rezaei, 2022).

Vaccinia virus (VACV) protects against smallpox and monkeypox, as VACV-based vaccines are among the most successful vaccines developed and closely resemble other PXVs, suggesting significant potential for developing treatments. VACV is characterized by its ability to accommodate large gene insertions, making it also a promising oncolytic agent (Hsu et al., 2024). Pexa-Vec (Pexastimogene Devacirepvec; JX-594, TG6006) is a genetically modified vaccinia virus in which the thymidine kinase gene, an enzyme involved in DNA synthesis, has been deleted. This modification enhances the virus’s selectivity for tumor cells, which typically exhibit elevated levels of thymidine kinase activity. Other advantages include GM-CSF expression, which increases tumor infiltration by cytotoxic T lymphocytes, antibody-mediated cytotoxicity, and complement activation (Samson et al., 2022).

#### 2.4.4 Rhabdoviruses and measles virus

Belonging to the Rhabdoviridae family, the vesicular stomatitis virus (VSV) is also known as an arbovirus and ranges in size from 65 to 185 nm, carrying a negative-strand RNA genome. VSV has tropisms for tumor cells deficient in interferon type 1, one factor that makes the infection more specific. In addition to this specificity, alterations are also made to the genetic material and its regulatory elements, such as the deletion of viral virulence genes, insertion of therapeutic genes, such as the thymidine kinase gene of herpesvirus, and expression of cytokines that stimulate the immune system (Ahmed et al., 2024).

Measles virus (MV) belongs to the Paramyxoviridae family and is a highly contagious human pathogen with tropism for lymphocytes and epithelial cells. MV can also enter the central nervous system, causing serious complications. It also has a negative-sense RNA strand, and its genome is approximately 16 kb in size (Amurri et al., 2022; Engeland and Ungerechts, 2021). Attenuated vaccine strains of MV have been shown to exhibit naturally oncolytic properties when CD46 is expressed in tumor cells, while replicating more readily in cells with a deficient IFN-I response, inducing immunogenic death of tumor cells, maturation of dendritic cells, and cross-presentation of tumor antigens (Chatela et al., 2024).

#### 2.4.5 Enterovirus: coxsackievírus A21 (CVA21)

Enteroviruses have single-stranded RNA genomes, are icosahedral, non-enveloped, and measure 30-32 nm in size. These viruses exhibit considerable environmental stability and may remain viable at room temperature for several days. Enteroviruses are associated with millions of infections worldwide each year, predominantly affecting children under 5 years of age (Gonzalez et al., 2019).

Coxsackievirus A21 (CVA21) belongs to the Picornaviridae family and the genus *Enterovirus*. It is used as an oncolytic agent that selectively targets cells expressing intercellular adhesion molecule-1 (ICAM-1) and decay-accelerating factor (DAF), a glycosylphosphatidylinositol-anchored glycoprotein (Annels et al., 2018). The expression of these molecules enables the selective infection of cells by this virus, particularly in solid tumors such as Melanoma (Shafren et al., 1997), including some breast cancer cell lines (Skelding et al., 2009) and prostate cancer cell lines (Berry et al., 2008).

#### 2.4.6 Reovirus: pelareorep and JIN-3

Reoviruses such as Pelareorep and JIN-3 contain double-stranded RNA as their genetic material. The presence of double-stranded RNA is a hallmark of many viral infections, both because it constitutes the genome of certain viruses and because it can be generated as an intermediate during viral replication and protein synthesis, thereby triggering innate immune recognition and activating both intrinsic and extrinsic cellular antiviral responses. In addition, mammalian cells may also produce double-stranded RNA under conditions of dysregulation, eliciting similar immune signaling pathways (Chen and Hur, 2022).

Reovirus selectivity for tumor cells is attributed to the upregulation of genes involved in the Ras signaling pathway. In normal cells, the detection of viral double-stranded RNA induces the expression and activation of protein kinase R (PKR), thereby inhibiting viral protein translation. In contrast, in many tumor cells, oncogenic alterations that activate Ras signaling can impair PKR-mediated phosphorylation and downstream antiviral responses, resulting in the loss of translational control and facilitating viral replication (Vidal et al., 2008).

Pelareorep is an oncolytic, non-enveloped, double-stranded RNA virus. A mutation in the *KRAS* gene can selectively lyse colorectal cancer cells by promoting viral propagation, inducing apoptosis, and increasing cell death *via* the expression of apoptotic biomarkers (Jiffry et al., 2021).

The oncolytic virus JIN-3, a mutant reovirus that is independent of junctional adhesion molecules, has recently been evaluated in preclinical models of human urothelial bladder carcinoma. It induces tumor cell lysis like the wild-type strain, but with greater efficacy in a dose-dependent manner, mediated by increased expression of interferon-stimulated genes, cell death markers, and pro-inflammatory cytokines. In addition, it exhibits enhanced tropism for tumor cells, making it a promising candidate for clinical trials (van de Merbel et al., 2026).

Table 1 shows a comparison of some viral platforms used for cancer treatment applications.

**TABLE 1:** Overview of selected viral platforms for oncolytic and cancer gene therapy applications.

Viral platform	Genome type	Immunogenicity	Main advantages	Main limitations	Clinical applications	Examples	References
Adenovirus	dsDNA	High	Selective tumor promoters, prodrug-activated cytotoxicity, and immunostimulatory payloads	Strong immune clearance	Cancer gene therapy; vaccines	CAN-2409 Ad5-CD/TKrep CG0070 Nadofaragene firadenovec	(Wang J. et al., 2022, Uchio et al., 2022)
Herpes simplex virus (HSV)	dsDNA	Moderate–High	Large payload capacity; neurotropism	Complex engineering	Oncolytic virotherapy	T-VEC Vusolimogene orparepvec RP1	(Li et al., 2022a, Li et al., 2022b)
Poxvirus	dsDNA	High	Cytoplasmic replication; large transgene insertion capacity; GM-CSF expression	High immunogenicity	Cancer immunotherapy	Pexa-vec (JX-594) Pexastimogene TG6006	(Wong et al., 2025–Hsu et al., 2024)
Rhabdovirus	ssRNA	Moderate	Rapid replication in tumors	Neurotoxicity concerns	Experimental oncolytic therapy	Vesicular stomatitis virus	(Samson et al., 2022)
Paramyxovirus	ssRNA	Moderate	Strong cell fusion activity; efficient tumor spread	Pre-existing immunity	Hematologic malignancies	Measles virus	(Engeland and Ungerechts, 2021)
Picornavirus	ssRNA	Moderate	Natural tropism for tumor cells	Limited cargo capacity	Solid tumors	Coxsackievírus A21	(Gonzalez et al., 2019, Annels et al., 2019)
Reovirus	dsRNA	Low–Moderate	Selective replication in ras-activated cells	Limited genetic engineering	Solid and metastatic tumors	Pelareorep *Jin-3*	(Berry et al., 2008, Chen et al., 2022)

## 3 Oncolytic virus platforms in genitourinary cancers

The power of OVs, in addition to their ability to lyse tumor cells, also lies in the precision with which they were engineered. Adenoviruses, HSV, PXVs, VSV, reovirus, and MVs have shown promising preclinical and clinical activity in prostate, bladder, and renal cancers (Harrington et al., 2019; Gujar et al., 2018).

One of the defining characteristics of GU tumors, especially PC and BC, is their poor immunogenicity. As a result, while ICIs were shown to be transformative in melanoma and lung cancer treatment, they have yielded disappointing results for many GU malignancies. For instance, the KEYNOTE-199 trial of pembrolizumab in metastatic CRPC reported an objective response rate of <5%, highlighting the need for novel immune-priming strategies (Antonarakis et al., 2020). Since OVs are uniquely positioned to meet this challenge, for example, by activating pattern recognition receptors, such as toll-like receptors and *RIG-I* (retinoic acid-inducible gene I), among other mechanisms, this reprogramming may result in local and systemic immune activation (Shimabukuro-Vornhagen et al., 2012; Lindau et al., 2013).

Another strength of OV therapy in GU cancers lies in the route of administration. Tumors such as the bladder and prostate are readily accessible *via* transurethral or transrectal approaches, enabling intratumoral injection. This strategy can maximize viral replication and immune stimulation while minimizing off-target toxicity (Monge et al., 2023; Burke et al., 2012).

### 3.1 Oncolytic viruses in prostate cancer

#### 3.1.1 Tumor characteristics and immunoresistance

PC present distinct immunological challenges that have rendered conventional immunotherapies largely ineffective. Following the broad immunological framework established by Chen and Mellman (2017), which categorizes tumors based on their immune infiltration patterns, prostate tumors are predominantly characterized as “immune deserts” (Chen and Mellman, 2017). As recently detailed by Novysedlak and colleagues (Novysedlak et al., 2024), prostate tumors are often characterized as “immunologically cold”; this “cold” status reflects several converging mechanisms of immune resistance that allow the tumor to remain invisible to the host’s defenses. A low tumor mutational burden and limited neo-antigen diversity fundamentally impair the immune system’s capacity to recognize and present malignant cells for elimination. Antigen presentation deficits are further exacerbated by downregulation of MHC-I molecules, which prevents effective priming of the immune response. In addition, the expression of cytokines and chemokines that dampen immune activation contributes to this suppressive ecosystem (Novysedlak et al., 2024). Although genitourinary malignancies share several immunosuppressive mechanisms, distinct pathways may predominate depending on the tumor type. In prostate cancer, for instance, high expression of *B7-H3* has been frequently reported. B7-H3 is a transmembrane protein that functions as an immune checkpoint molecule involved in the regulation of T Cell activation and antitumor immune responses. In addition to its immunomodulatory role, B7-H3 has been associated with tumor cell proliferation, migration, adhesion, signal transduction, metastasis, and therapeutic resistance. Notably, elevated B7-H3 expression has also been observed in renal cell carcinoma (Shen et al., 2024).

Although androgen deprivation therapy, chemotherapy, and next-generation hormonal agents have significantly advanced the clinical prognosis in both localized and metastatic settings, the marginal efficacy of ICIs underscores a critical need for strategies capable of dismantling the immunosuppressive barriers inherent to prostate malignancies. This therapeutic gap was notably evidenced in the phase II KEYNOTE-199 trial; despite the use of pembrolizumab in patients with metastatic castration-resistant prostate cancer, the study yielded only modest objective response rates. Such outcomes highlight the intrinsic resistance of PC to current checkpoint blockade protocols and the need to develop novel interventions to overcome this immunological stagnation (Antonarakis et al., 2020).

Given these barriers, therapeutic approaches capable of both directly lysing tumor cells and reprogramming the TME are urgently needed. OVs are uniquely positioned to exert dual mechanisms of action; they not only facilitate the immediate reduction of tumor mass but also initiate a robust systemic anti-tumor immune response, often described as *in situ* vaccination (Kaufman et al., 2015; Lichty et al., 2014). The following sections examine the specific immunobiological hurdles characteristic of PC and highlight emerging OV platforms designed to overcome them (Kaufman et al., 2015; Lichty et al., 2014).

#### 3.1.2 CAN-2409

CAN-2409 (Aglatimagene Besadenovec) is a non-replicating adenoviral vector engineered to deliver the herpes simplex virus thymidine kinase (HSV-TK) gene directly into tumor cells (Herman et al., 1999; Wheeler et al., 2016). This gene-mediated cytotoxic immunotherapy platform operates *via* a suicide gene mechanism: upon administration of antiviral prodrugs such as acyclovir, ganciclovir, or valacyclovir, the HSV-TK enzyme converts the prodrug into toxic metabolites that terminate DNA synthesis (Wheeler et al., 2016). This process induces ICD, effectively releasing tumor-associated antigens and proinflammatory signals that prime the systemic immune system (Herman et al., 1999; Wen et al., 2025).

A phase III clinical trial involving 745 patients used treatment for localized PC of intermediate to high risk. The trial involved combining CAN-2409 or a placebo with external beam radiotherapy concomitantly with valacyclovir, in the presence or absence of androgen deprivation therapy. After a median follow-up exceeding 50 months, treatment with CAN-2409 resulted in a 30% reduction in the risk of recurrence or death compared to the placebo group. Furthermore, biopsies performed 2 years after treatment showed an 80.4% pathologic complete response in the CAN-2409 group, compared with 63.6% in the control group. The regimen was well tolerated, with adverse events primarily limited to low-grade flu-like symptoms (DeWeese et al., 2025). While this platform has also shown efficacy in other solid tumors such as glioma (Wheeler et al., 2016; Wen et al., 2025), the Phase III prostate cancer data represent the most significant clinical milestone to date.

#### 3.1.3 Ad5-CD/TKrep

In three clinical studies of recurrent PC, Freytag and colleagues worked with live, replicating adenovirus (Ad5). This virus was engineered to deliver a suicide gene fusion of cytosine deaminase/thymidine kinase from herpes simplex virus type 1 (CD/TKrep) to the cell, making the tumor sensitive to pharmacological agents and radiation. The phase I clinical study consisted of intraprostatic application of the virus, followed by 5-fluorocytosine and ganciclovir prodrug applications after 2 days 94% of the adverse effects observed were classified as mild to moderate; in addition, other promising results included a decrease in PSA levels in 63% of patients (Freytag et al., 2007a). Another study followed these same patients for 5 years, analyzing serum PSA levels and biopsies after treatment, showing that the suicide gene therapy performed had a long-term benefit by delaying the increase in PSA and the eventual need for androgen deprivation therapy, which is associated with reduced quality of life and substantial patient morbidity. Additionally, a predictive model demonstrated that the requirement for salvage therapy could be postponed by up to 2 years (Freytag et al., 2007b).

#### 3.1.4 Other OV approaches

Pelareorep (Reolysin) is a naturally non-attenuated reovirus (Dearing strain) that exploits the activated Ras signaling pathways, predominantly found in malignant cells. While it remains non-pathogenic to healthy tissues, the virus selectively replicates within Ras-activated cancer cells, inducing targeted oncolysis and potent cytotoxic effects (Mahalingam et al., 2017; Chakrabarty et al., 2015).

An early-phase investigation involving patients with localized PC evaluated the safety of intratumoral Pelareorep administration before radical prostatectomy. The regimen was well tolerated, and subsequent histopathological analyses revealed significant CD8^+^ T Cell infiltration and enhanced tumor apoptosis (Thirukkumaran et al., 2010). Conversely, the CCTG IND 209 phase II trial assessed the efficacy of intravenous Pelareorep in patients with metastatic castration-resistant prostate cancer. Participants were randomized to receive docetaxel and prednisone either in combination with Pelareorep (n = 41) or as monotherapy (n = 44), with the primary endpoint defined as the disease progression rate. Although the combination maintained a favorable profile, it failed to improve clinical outcomes; no statistically significant differences in progression-free survival or overall survival were observed compared to the control arm. These results suggest that Pelareorep’s limited efficacy in this setting may be due to suboptimal synergy with cytotoxic chemotherapy. Another hypothesis might be the administration route of choice. Nevertheless, future research should explore its potential as a neoadjuvant immune primer before radiotherapy or surgery or investigate its combination with ICIs (Eigl et al., 2018).

### 3.2 Oncolytic viruses in bladder cancer

#### 3.2.1 Tumor background and need for alternatives

Bladder cancer, particularly NMIBC, represents a frequent and recurrent malignant neoplasm (70,71). Failure of intravesical BCG therapy, although effective in many patients, remains a significant clinical challenge and can lead to the development of persistent or recurrent disease. Radical cystectomy, however, carries substantial risks, including urinary diversion, sexual dysfunction, and perioperative complications, and is particularly expensive for elderly patients or those with comorbidities, reducing their quality of life, especially in the first 5 years after surgery (Grabe-Heyne et al., 2023). This therapeutic gap has catalyzed the pursuit of bladder-sparing alternatives, especially in combination with other treatment modalities, including ICIs, immunotherapy, radiotherapy, and chemotherapy (Hu et al., 2022). Urothelial bladder carcinoma, similar to renal cell carcinoma, is characterized by a high mutational burden. The increased expression of neoantigens resulting from this elevated mutational load has been positively associated with improved responses to immune checkpoint inhibitor therapies, including anti-PD-1 and anti-CTLA-4 agents. Despite this profile, the tumor microenvironment remains profoundly immunosuppressive, with substantial infiltration by myeloid-derived suppressor cells and tumor-associated macrophages, increased prostaglandin E2 production, and dysregulated glycosaminoglycan metabolism. Collectively, these factors contribute to the recruitment and expansion of regulatory T Cells, thereby promoting immune evasion and tumor progression (C et al., 2020).

#### 3.2.2 Cretostimogene Grenadenorepvec

CG0070 (Cretostimogene Grenadenorepvec) is among the most advanced oncolytic viruses under investigation for NMIBC in patients who do not respond to BCG treatment. This is a conditionally replicating oncolytic serotype 5 adenovirus, genetically modified with the addition of the *F2F1* gene, a tumor-specific transcription regulator that has deficits in the retinoblastoma pathway (cell cycle control, as in most bladder neoplasms) while also inhibiting the expression of the viral genes E1A, which block viral replication and cytotoxicity. In this approach, the virus becomes selective, as it exhibits tropism for tumor cells that do not restrict its replication and virulence mechanisms. Importantly, CG0070 also encodes GM-CSF, initiating anti-tumor immune responses following the release of tumoral antigens (Ramesh et al., 2006; Li R. et al., 2022).

In a phase I trial, patients with NMIBC (n = 35) received intravesical infusions of CG0070 across different dosing cohorts. Most adverse events were grade 1 or 2, with dysuria being the most frequently reported. Evidence of antitumor activity was also observed: the complete response rate among all patients was 48.6% at 10.4 months, 63.6% in patients who received multiple doses, and 81.8% in patients with borderline or high retinoblastoma phosphorylation who received multiple doses (Burke et al., 2012).

Combination therapies with ICIs are also promising. In the phase II CORE-001 trial, CG0070 was administered in combination with pembrolizumab (anti-PD-1 mAb) in patients with BCG-unresponsive NMIBC with carcinoma *in situ* (n = 35). The combination regimen was well tolerated and demonstrated a favorable benefit–risk profile, with CR of 77.1%, 54.3%, and 51.4% at 6, 8, and 24 months, respectively (Li et al., 2024). These results place CG0070 at the forefront of bladder-sparing strategies for patients with limited treatment options.

#### 3.2.3 Nadofaragene Firadenovec

Nadofaragene firadenovec-vncg (rAd-IFNα/Syn3) represents a significant milestone in the clinical translation of gene-based intravesical therapies. The administration of this recombinant adenoviral vector encoding the human interferon alpha-2b gene to urothelial and bladder tumor cells results in IFN-α expression, with immunostimulatory, antiangiogenic, and apoptotic effects. This is combined with the use of a compound, Syn3, which helps remove the glycosaminoglycan layer from the bladder lining, facilitating viral access (Steinmetz et al., 2024).

The pivotal phase III study (NCT02773849) that supported the subsequent FDA approval enrolled patients (n = 151) with high-grade Ta/T1 NMIBC who were BCG-unresponsive. Complete response (CR) was observed in 53% of patients with carcinoma *in situ*, with or without concomitant Ta/T1 disease, at the 3-month time point, and 45.5% of responders remained disease-free at 12 months. The therapy was generally well tolerated, with the most common adverse event being micturition urgency, and only 2 grade 3–4 events were related (Boorjian et al., 2021). More recently, a study approved by the Mayo Clinic Institutional Review Board studied 46 patients with NMIBC unresponsive to BCG therapy who received Nadofaragene Firadenovec from November 2023 through December 2024 at Mayo Clinic. The primary outcome was the patient’s CR at any time point during the trial (when cystoscopy and urine cytology were negative or atypical), and CRs were included after 3 and 6 months (after reinduction with Nadofaragene Firadenovec for patients who persisted after the first 3 months). The estimated CR rate at 6 and 12 months for patients with carcinoma *in situ* was 67% and 54%, respectively (Moyer et al., 2026).

Taken together, the clinical success of CG0070 and Nadofaragene Firadenovec underscores the therapeutic potential of exploiting cancer cell susceptibility to viral mechanisms in conjunction with localized immunostimulation. By addressing tumor recurrence and immune evasion through innovative vector design, these agents offer patients with BC not only meaningful clinical benefits but also the opportunity to preserve bladder function and quality of life.

#### 3.2.4 Coxsackievírus A21 (CVA21)

Coxsackievirus A21 (CVA21) is an oncolytic agent that infects cells expressing intercellular adhesion molecule-1 (ICAM-1) and the decay-accelerating factor (DAF) glycosylphosphatidylinositol-anchored glycoprotein (Annels et al., 2018). The formulation known as CAVATAK contains Coxsackievirus A21 and has demonstrated immunotherapeutic efficacy *in vitro*, in animal models, and in human clinical trials when administered intratumorally. Furthermore, it may also be used in combination with immune checkpoint inhibitors (Annels et al., 2018; Annels et al., 2019).

A study conducted by Annels and colleagues (Annels et al., 2018) demonstrated the efficacy of coxsackievirus A21 in treating non-muscle-invasive bladder cancer in cell cultures and tissue samples, and the potential to modulate the expression of the viral receptor ICAM-1. In addition to its ability to induce tumor apoptosis, it releases DAMPs, thus stimulating immunogenic cell death. These findings provided the rationale for a phase I clinical trial investigating this therapeutic strategy in combination with agents such as immune checkpoint inhibitors.

In this clinical study, fifteen patients with NMIBC were enrolled prior to surgical resection and allocated into two groups: nine patients received CAVATAK, while the remaining six received CAVATAK in combination with a subtherapeutic dose of mitomycin C, which is known to enhance ICAM-1 expression in bladder cancer cells. No significant adverse effects were observed at the administered doses. Although mitomycin C increased viral uptake, it did not alter CD8^+^ T Cell infiltration within the tumor in this study. Analysis of biopsies from surgically resected tumors revealed induction of intratumoral inflammation and hemorrhage, associated with upregulation of interferon inducible genes, including programmed death-ligand 1 (PD-L1) and T helper 1 (Th1)-associated chemokines, among other immune-related factors, compared with untreated patients (Annels et al., 2019).

#### 3.2.5 Reovirus: pelareorep and JIN-3

Reoviruses, including Pelareorep and JIN-3, have double-stranded RNA (dsRNA) genomes. The recognition of dsRNA is commonly associated with viral infections, both because it constitutes the genetic material of several viral species and because it is generated during viral replication and protein synthesis. The presence of dsRNA activates innate immune surveillance mechanisms that identify this molecular pattern as a potential threat and, consequently, induce intrinsic and extrinsic cellular responses aimed at controlling infection. Furthermore, mammalian cells can produce endogenous dsRNA under dysregulated physiological conditions, thereby activating similar immune signaling pathways and promoting antiviral immune responses (Chen and Hur, 2022).

Reovirus selectivity for tumor cells is associated with the upregulation of genes involved in the Ras signaling pathway. In normal cells, the detection of viral double-stranded RNA induces activation of protein kinase R, which inhibits viral protein translation and limits viral replication. In many tumor cells, however, oncogenic alterations leading to constitutive Ras pathway activation can impair PKR activation and downstream signaling, thereby disrupting antiviral translational control and permitting efficient viral replication (Vidal et al., 2008).

Pelareorep is an oncolytic, non-enveloped, double-stranded RNA virus. A mutation in the *KRAS* gene can selectively lyse colorectal cancer cells by promoting viral propagation, inducing apoptosis, and increasing cell death *via* the expression of apoptotic biomarkers (Jiffry et al., 2021).

The oncolytic virus JIN-3, a mutant reovirus independent of junctional adhesion molecules, has recently been evaluated in preclinical models of human urothelial bladder carcinoma. Like the wild-type strain, JIN-3 induces tumor cell lysis; however, it demonstrates greater efficacy in a dose-dependent manner, associated with increased expression of interferon-stimulated genes, cell death markers, and inflammatory cytokines. Furthermore, JIN-3 exhibits enhanced tropism for tumor cells, highlighting its potential as a promising candidate for future clinical trials (van de Merbel et al., 2026).

### 3.3 Oncolytic viruses in renal cell carcinoma

#### 3.3.1 Renal cell carcinoma (RCC) immunogenicity and the TME

RCC, particularly the clear cell subtype (ccRCC), is distinct among solid tumors due to its pronounced immunogenicity and extensive vascularization. Clear cell renal cell carcinoma is considered a highly immunogenic tumor. Nevertheless, it can also promote immune dysfunction by recruiting and infiltrating immunosuppressive cell populations, particularly regulatory T Cells, thereby contributing to tumor immune evasion (Choueiri and Kaelin, 2024; Díaz-Montero et al., 2020). In addition to this characteristic, these tumors frequently harbor mutations that inactivate both alleles of the von Hippel–Lindau (*VHL*) gene, which encodes pVHL, a component of the E3 ubiquitin ligase complex responsible for oxygen sensing. Under normal oxygen conditions, the E3 ubiquitin ligase complex promotes the degradation of hypoxia-inducible factor (HIF), thereby suppressing its activity. However, mutations in *VHL* result in the accumulation of HIF, which subsequently upregulates the expression of hypoxia-responsive genes, including *VEGFA*, which encodes VEGF (vascular endothelial growth factor), thereby promoting tumor angiogenesis (Choueiri and Kaelin, 2024).

This genetic alteration leads to the overexpression of angiogenic factors and profound reprogramming of the TME immune landscape. Although the ccRCC TME is highly infiltrated and exhibits a proinflammatory profile, T Cell responses are frequently dysfunctional, contributing to the limited efficacy of anti-PD-1 therapy. Among the mechanisms underlying this immune dysregulation, cytokine release by proinflammatory tumor-associated macrophages is thought to drive the hampering of the T Cell response (Wolf et al., 2024; Mandriota et al., 2002). In addition to CD8^+^ T Cell inhibition, NK cells also undergo alterations in TME infiltration, activation, and functional profiles following VHL loss. Conversely, the restoration of VHL activity has been shown to suppress tumor progression and alleviate immune suppression, as evidenced by the deregulation of MHC class I and PD-1 (Wolf et al., 2024; Tong et al., 2025; Jiao et al., 2024).

While ICI therapy has increased the survival rates of RCC, resistance remains a challenge (Lasorsa et al., 2023; Chung et al., 2025). Within this context, OVs emerge as a promising adjunct. By selectively infecting and lysing tumor cells, OVs exert direct cytotoxicity and facilitate the release of tumor associated antigen, PAMPs and DAMPs, which initiate and amplify local immune responses. Engineered OVs can further enhance this immunogenic cascade by delivering transgenes encoding immunostimulatory cytokines (e.g., GM-CSF, IL-12) or costimulatory molecules that reprogram the immune response (Wu et al., 2023; Palanivelu et al., 2023; Ma et al., 2020).

#### 3.3.2 Pexa-Vec in renal cell carcinoma

Around 20%-30% of patients with RCC respond to ICI treatments; to fill this gap, more effective treatment methods are being developed (Park et al., 2025). Pexa-Vec (JX-594) is an immune-oncolytic therapy that aims to transform non-inflamed tumors into inflamed tumors, thereby stimulating the influx of cells capable of recognizing and eliminating tumor cells (Monge et al., 2023). This virus has been engineered to insert the human *GM-CSF* and *LacZ* genes into the thymidine kinase gene, conferring oncolytic activity (Pa et al., 2022; Bartlett et al., 2013; Breitbach et al., 2015).

In murine xenotransplantation models of ccRCC, intratumoral administration of Pexa-Vec resulted in significant tumor reduction and increased interferon-β expression. (Park et al., 2025). Similarly, in metastatic orthotopic RCC models, intraperitoneal administration reduced primary tumor burden and lung metastases in both early- and advanced-stage disease, concomitant with reprogramming of the TME immune response (Pa et al., 2022). In a murine orthotopic metastatic RCC model, the combination of a PD-1 inhibitor with Pexa-Vec reduced the primary tumor and metastases in early stages of the disease, compared with the combination of ICIs (anti-PD-1 and CTLA-4), through TME remodeling. However, in advanced stages, there was no difference in tumor size between the two treatments, although treatment with Pexa-Vec and PD-1 inhibitor showed diminished liver toxicity (Park et al., 2024).

An ongoing phase Ib/IIa clinical trial evaluating intravenous or intratumoral administration of Pexa-Vec in combination with Cemiplimab (anti-PD-1 mAb) is currently active and involves patients with metastatic or unresectable ccRCC (NCT03294083). This reprogramming effect, mediated by the induction of inflammatory chemokines, DC recruitment, and reversal of myeloid immune suppression, suggests that Pexa-Vec, and potentially, OVs may serve best as immunological primes for combination regimens rather than standalone agents, as demonstrated in a murine model (Pa et al., 2022; Park et al., 2024).

Table 2 summarizes the main types of OVs studied in genitourinary cancer, their engineering strategies, and therapeutic use.

**TABLE 2:** Oncolytic virus platforms in clinical trials of genitourinary tumors.

Virus type	Development stage and year of publication	Mechanism of action	Genetic engineering	Application in GU tumors	Results	Limitations	Efficacy comparison	References
CAN-2409 (adenovirus)	Phase III clinical trial (2025)	Intratumoral delivery of HSV-tk	Herpes virus thymidine kinase gene into an adenoviral vector	Localized PC	−30% reduction in recurrence or death (50 months) - CR increase of 16.8% (2 years)	- Intraprostatic administration - Mostly grade 1 and 2 adverse effects (1.7% of serious treatment associated reports)	Ad5-CD/TKrep and CAN-2409 were shown to be safe, associated with improved survival. In contrast, the combination of pelareorep with docetaxel failed to enhance the efficacy of chemotherapy	(DeWeese et al., 2025)
Pelareorep (reovirus)	Phase II clinical trial (2018)	Activation of ras signaling pathways	Natural non-attenuated reovirus (dearing strain)	Metastatic castration resistant PC	No statistically significant differences were observed in progression-free or overall survival when compared to monotherapy	- Intravenous administration - Increase in adverse events in coadministered group (mostly grade 1 and 2, but also grade 3)		(Eigl et al., 2018)
Ad5-CD/TKrep (adenovirus)	Phase I clinical trial (2007)	Restore tumor sensitivity to pharmacological agents and radiation	Delivery a suicide gene fusion of cytosine deaminase/thymidine kinase from HSV type 1 into a live Ad5	Localized PC	- Decrease in PSA levels of 63% of patients (2 years) - Postponement of salvage therapy - Increase in overall survival time	- Intraprostatic administration - Mostly (93%) adverse effects observed were classified as mild to moderate (only grade 3 or worse adverse effect associated with OV therapy was lymphopenia and hyperglycemia)		(Freytag et al., 2007a, Freytag et al., 2007b)
CG0070 (adenovirus)	Phase I clinical trial (2012)	-Selective replication in retinoblastoma-defective tumors -Activation of dendritic cells and the anti-tumor immune response	Expression of F2F-1 + GM-CSF using an Ad5 platform	NMIBC (carcinoma *in situ*) after BCG failure	- CR of 48.6% (10.4 months) - CR increases to 63.6% in patients who received multiple doses −81.8% CR in patients with borderline or high retinoblastoma phosphorylation + multiple doses	- Mostly grade 1 and 2 adverse effects (3 out 35 patients showed grade 3 or worse adverse events)	- Both OVs demonstrated safe and promising results, increasing CR up to 24 months - Administration of CG0070 was able to inhibit progression to invasive disease	(Monge et al., 2023)
CG0070 (adenovirus)	Phase II clinical trial (2024)	- Selective replication in retinoblastoma-defective tumors - Activation of dendritic cells and the anti-tumor immune response	Expression of F2F-1 + GM-CSF using an Ad5 platform	NMIBC carcinoma *in situ* after BCG failure	−51.4% CR rate at 24 months - No patient progressed to MIBC - CR of 77.1%, 54.3%, and 51.4% at 6, 8, and 24 months, respectively	- Most adverse events were grade 1 or 2 (mainly bladder-associated symptoms). No grade 3 or above adverse events attributed to CG0070 were reported		(Li et al., 2024)
Nadofaragene firadenovec (adenovirus)	Phase III clinical trial (2021)	- Stimulation of immune response and apoptosis - Angiogenesis inhibition	Expression of human IFN-α2b gene + Syn3 excipient	High-grade NMIBC patients unresponsive to BCG therapy	- CR of 53% and 45.5% over 3 and 12 months, respectively	- Mild adverse events. Mostly grade 1 and 2 (66%), with 4% of grade 3 events. No report of events grade 4 or above		(Boorjian et al., 2021)
Coxsackievirus A2 - CVA21 (picornavirus)	Phase I clinical trial (2019)	- Immunogenic cell death induction of cells expressing intercellular adhesion molecule-1 and degradation-accelerating factor	- Natural virus CVA21, with or without a subtherapeutic dose of mitomycin C	NMIBC prior to surgery	- Induction of tumor inflammation and hemorrhage - No significant toxicities were reported in any patient	Mitomycin C did not confer any additional benefit to CVA21 treatment at the dose used		(Annels et al., 2019)
Nadofaragene firadenovec (adenovirus)	Retrospective cohort study (2026)	- Stimulation of immune response and apoptosis - Angiogenesis inhibition	Expression of human IFN-α2b gene + Syn3 excipient	NMIBC patients unresponsive to BCG therapy	- CR in the carcinoma *in situ* cohort: 67% and 54% at 6 and 12 months, respectively	- Grade 1 or 2 adverse events. Only 9% grade 3 adverse events. No grade 4 or 5 adverse effects were		(Moyer et al., 2026)

## 4 Challenges and limitations in ov-based therapies

### 4.1 Delivery barriers

One of the most persistent barriers to the effective implementation of oncolytic virotherapy is the challenge of delivering the virus to tumor sites in a manner that allows sufficient replication without premature neutralization. Systemic administration is an appealing treatment for disseminated or inaccessible metastases; however, it often suffers from rapid clearance by the host immune system, especially in patients with pre-existing antiviral antibodies from natural infection or prior vaccinations. For instance, clinical trials of vaccinia and adenovirus-based vectors have demonstrated a significant reduction in viral persistence in circulation, thereby limiting their capacity to establish productive infection in tumors (Xia et al., 2025; Wu et al., 2025). Conversely, intratumoral injections have been shown to produce high local concentrations of viruses, ensuring direct infection of tumor cells (De Lombaerde et al., 2021). However, logistical constraints in the context of deep lesions, such as renal lesions, are particularly concerning, as image-guided injections are imperative (Xia et al., 2025).

Pre-existing antiviral immunity represents a double-edged sword in oncolytic virotherapy. Neutralizing antibodies, complement activation, and rapid uptake by phagocytic cells can limit systemic viral delivery and reduce therapeutic efficacy, particularly for widely circulating viruses such as human adenovirus (Workenhe and Mossman, 2014; Marelli et al., 2018; Kuryk and Møller, 2023). However, pre-existing immunity does not necessarily abrogate therapeutic benefit. Viral infection within the tumor can still promote local immune activation and antigen release. Antiviral cytotoxic CD8^+^ T Cells recognizing viral epitopes expressed in infected tumor cells may contribute to tumor killing, while virus-induced immunogenic cell death leads to the release of damage-associated molecular patterns (DAMPs) and tumor-associated antigens. This process can enhance dendritic cell activation and promote cross-priming of tumor-specific T Cell responses, thereby amplifying systemic antitumor immunity (Workenhe and Mossman, 2014; Gauvrit et al., 2008). Increasingly, patient stratification strategies are being explored to optimize OV selection. Factors such as baseline antiviral antibody titers, tumor accessibility for intratumoral administration, expression of viral entry receptors (e.g., CAR for adenovirus or CD46 for measles virus), and defects in antiviral signaling pathways such as type I interferon responses may help identify patients most likely to benefit from a given OV platform (Harrington et al., 2019; Chiocca and Rabkin, 2014). In this context, matching viral tropism and replication requirements with tumor molecular features and host immune status may represent an important step toward precision viro-immunotherapy.

To overcome these barriers, researchers have employed a variety of strategies. The encapsulation of viruses in liposomes or polymeric nanoparticles has demonstrated efficacy in preclinical studies, protecting viral particles from neutralizing antibodies and prolonging circulation time (Yan et al., 2025). In a similar manner, carrier-cell approaches wherein viruses are delivered using mesenchymal stem cells or activated T Cells provide a “Trojan horse' strategy to bypass systemic immune clearance and selectively home to tumors (Xia et al., 2025; Bell and Roy, 2013). Collectively, these findings illustrate that no single approach is universally effective; rather, future OV therapy will likely require hybrid delivery strategies that balance systemic reach with localized potency, tailored to tumor accessibility and disease burden.

### 4.2 Tumor selectivity and safety

A primary conceptual advantage of OVs is their ability to replicate selectively within malignant tissues while sparing normal cells. This selectivity is typically mediated through binding to receptors expressed on the surface of tumor cells, which are frequently overexpressed during tumorigenesis, thereby facilitating viral internalization. The principal receptors involved in this process include CAR for adenovirus and coxsackievirus; ICAM-1 and DAF for coxsackievirus; signaling lymphocytic activation molecule (SLAM) and CD46 for measles virus; sialic acid receptors and junctional adhesion molecule A (JAM-A) for reovirus; herpesvirus entry mediator (HVEM), nectin-1, and nectin-2 for herpesvirus; and the low-density lipoprotein receptor for vesicular stomatitis virus. Interestingly, vaccinia virus does not require a specific cellular receptor for entry (Xiao et al., 2026). Selectivity may also be engineered by attenuating the virus through the deletion of genes essential for replication in post-mitotic normal cells but dispensable in the nutrient-rich, proliferative environment of tumor cells. Alternatively, tumor-specific promoters may be utilized to drive the expression of critical viral genes. However, achieving absolute tumor exclusivity remains a significant challenge (Xiao et al., 2026; Martuza, 2000).

Early clinical investigation using HSV-derived OVs, notably the G207 mutant, reported occasional off-target infection of non-malignant tissues, which raised critical concerns regarding neurotoxicity and unintended viral spread (Hunter et al., 1999). Similarly, the systemic administration of vaccinia-based vectors (such as JX-594) has, in rare instances, led to unintended replication in non-target tissues with high proliferative activity, such as skin and mucosal surfaces (Heo et al., 2013).

The risk of reversion or uncontrolled replication remains a critical concern, particularly in immunocompromised patients. Although rationally engineered designs, such as logic-gated viral constructs that require multiple tumor-specific molecular signals to initiate replication, have significantly enhanced safety profiles, continuous long-term vigilance is required. For instance, pivotal clinical studies with T-VEC in patients with advanced melanoma demonstrated that while the therapy was generally well-tolerated, a minority of participants developed constitutional “flu-like” symptoms. Furthermore, viral dissemination to untreated skin lesions was observed, highlighting the complex dynamics of viral spread and systemic immune activation (Andtbacka et al., 2015). These observations emphasize that improving safety does not simply entail more stringent replication restriction but also involves the engineering of layered safeguards that preserve therapeutic efficacy without compromising the patient. The current trajectory of OV research suggests that multiplexed genetic safeguards and precise, tumor-targeted regulation will increasingly define the balance between potency and safety. This evolution aims to facilitate selective tumor destruction while rigorously maintaining normal tissue integrity.

### 4.3 Immunological resistance in GU tumors

Although OVs are designed to exploit the host immune system, they paradoxically face barriers imposed by its antiviral immunity mechanisms of the host, such as the viral sequestration by splenic macrophages and hepatic Kupffer cells, following their opsonization, or neutralization *via* elements present in the serum, such as antibodies or proteins of the complement system (Russell et al., 2012). GU Tumor-induced antiviral responses include epigenetic factors, hindered tumoral cell infection within the TME due to early clearance by the host or extracellular production, the activity of global regulators (e.g., Sirtuin 1 (SIRT1)), and the secretion of immune signaling molecules that can trigger IFN-based responses (Evgin et al., 2015).

In a phase I trial with Pexa-Vec, for instance, 6 out of 23 patients with solid tumors increased neutralizing antibody production against vaccinia virus after only 15 days. However, antibody production did not correlate with antitumoral activity or therapy tolerance (Breitbach et al., 2011) To address this, reports with Pexa-Vec showed that the inhibition of the activation of complement was able to increase the serum OV concentration by an average of 10-fold in cynomolgus macaques, as well as increasing intratumoral OV concentration in a rat model, while also inhibiting viral neutralization with serum from cancer patients (Evgin et al., 2015). In another complement-focused approach, the construction of an OV based on the Pexa-Vec sequence, expressing human CD55 in its membrane proteins (SJ-607), was able to evade complement-mediated clearance, enhancing OV host survival in mice cancer models (Lee et al., 2023). Such mechanisms may prevent OVs from achieving the sustained replication required to reach and influence the TME, leading to treatment resistance and an inability to achieve the desired antitumoral effect in patients.

The idea of combining OVs with immune-targeted therapies, such as ICIs, is highly interesting due to the potential synergistic effects of immune stimulation combined with checkpoint blockade. VSV engineered to express the glycoprotein of lymphocytic choriomeningitis virus, and IL-12 has shown improved persistence in murine lung cancer models, where IL-12 promoted antitumor activity by generating long-lived effector TCD8^+^ cells (Hatami et al., 2025). In the CORE-001 trial, a phase 2 study with BCG-unresponsive NMIBC patients, Cretostimogene Grenadenorepvec (CG0070) was administered in combination with pembrolizumab. The combination was well tolerated, and no patient progressed to muscle-invasive disease. Additionally, the complete response rate at 2 years past treatment start was 51% (n = 35). Such combination options may open a possibility for bladder-sparing treatment options for BCG-unresponsive patients (Li et al., 2024).

Taken together, immunological resistance should be viewed as a dynamic process that requires rational design to overcome the host’s natural barriers and achieve optimal OV effectiveness.

### 4.4 Regulatory and manufacturing hurdles

While OV-based formulations have been shown to be well-tolerated by patients, without reports of recovery of viral pathogenicity, regulatory and manufacturing challenges remain, including overall production yield and delivery mechanisms (Russell et al., 2012). Unlike small molecules or monoclonal antibodies, OVs are live, replicating agents requiring highly controlled, large-scale good manufacturing practice production facilities. These particularities raise regulatory concerns, from preclinical trials through eventual approval, including the lack of standardized endpoints for evaluating success and the reproducibility of animal models (Xiao et al., 2026).

There is a long way to go before OVs are approved for clinical use. This reflects both the complexity of proving long-term efficacy and the cautious stance of regulatory agencies with therapies based on live viruses. Designs that incorporate focused on improved efficacy and specificity, such as mechanisms able to limit the viral infection exclusively to tumoral cells and safety kill-switches, may reassure regulatory bodies, however, extensive validation before approval is still required (Alwithenani et al., 2025). Thus, the field’s progress will depend not only on scientific innovation but also on the development of harmonized regulatory frameworks and scalable manufacturing pipelines that can keep pace with increasingly complex viral designs.

## 5 Translational applicability of OVs

### 5.1 Combination therapy

Although OVs hold considerable promise as novel therapeutic candidates across a wide range of tumors, their clinical implementation still faces several important challenges. Many of these barriers arise from intrinsic cancer resistance mechanisms, particularly the suppression of the host immune response and the presence of obstacles. These factors impair drug distribution and cell migration, largely due to the abnormal vasculature within the TME and the excessive deposition of extracellular matrix components (Chung et al., 2021). In addition, tissue remodeling is a hallmark of tumor progression, especially in PC. The composition and spatial organization of stromal and vascular cell populations, including fibroblasts, telocytes, pericytes, endothelial cells, smooth muscle cells, and neuronal cells, play a critical role in disease severity and progression (Assoni et al., 2026; Pederzoli et al., 2023).

To overcome these limitations, combination therapies have emerged as a promising strategy. The most common approach involving OVs has been their coadministration with ICIs. This rationale is based on OVs’ ability to induce ICD and apoptosis in tumors, which, when combined with immune checkpoint blockade, may help restore the host’s capacity to mount an effective antitumor response. The largest number of clinical trials evaluating this strategy has been conducted in melanoma, with variable outcomes. Notably, treatment with anti-PD-1 antibodies (nivolumab) in combination with intratumoral RP-1 (Vusolimogene Oderparepvec) in patients with advanced disease demonstrated encouraging results. This combination induced durable responses in patients who had failed ICI-based therapies, reduced lesion size, and improved survival outcomes previously. Additionally, phase I/II data indicated a favorable safety profile, with most adverse events classified as grade 1 or 2 ^76^. T-VEC was also evaluated in phase III trials in melanoma. While T-VEC monotherapy improved response rates and survival in patients with stage IIIB to IV disease in one study (Andtbacka et al., 2015), its combination with pembrolizumab did not yield additional benefit. Specifically, the OV + ICI regimen failed to improve progression-free survival or overall survival compared to pembrolizumab alone (Chesney et al., 2023). Another combination strategy involved the use of OVs with ICIs, specifically PexaVec in combination with Durvalumab (anti-PD-L1) and Tremelimumab (anti-CTLA-4) in patients with metastatic colorectal cancer who had previously received chemotherapy. In a phase I/II trial, this regimen was well tolerated, with only four patients (n = 34) requiring treatment interruption. The therapy was also associated with an increase in circulating CD8^+^ T Cell populations. Despite these favorable safety and immunological findings, progression-free survival was not significantly improved compared to other treatment approaches (Monge et al., 2023). Reports of OV and ICI combinations in GU tumors remain relatively limited. One example is the use of intravesical Cretostimogene Grenadenorepvec in combination with pembrolizumab in patients with NMIBC who had previously received BCG. This approach demonstrated a favorable safety profile and showed potential in preventing progression to muscle-invasive disease, thereby supporting the possibility of bladder-sparing treatment strategies (Li et al., 2024).

OVs have also been explored in combination with chemotherapeutic agents, based on evidence of potential synergy. Chemotherapy may enhance OV efficacy by inhibiting antiviral immune responses and facilitating viral entry and spread within the tumor. In line with this rationale, Pelareorep was evaluated in combination with carboplatin and paclitaxel in a phase II trial involving patients with advanced melanoma. The regimen was considered safe, with no grade 4 adverse events reported among the 14 enrolled patients. Disease control and objective response rates were 85% and 21%, respectively, while progression-free and overall survival outcomes were improved relative to established benchmarks (Mahalingam et al., 2017). In contrast, in patients with metastatic castration-resistant prostate cancer, intravenous administration of Pelareorep in combination with docetaxel did not result in improvements in progression-free survival or overall survival rates (Eigl et al., 2018).

Another combinatorial strategy involves integrating OVs with radiotherapy across different cancer types. OBP-301 has been evaluated in combination with radiotherapy in patients with esophageal cancer (Tanabe et al., 2021), while intratumoral RT3D (Reovirus Type 3 Dearing) has been administered in patients with advanced malignancies undergoing palliative radiotherapy (Harrington et al., 2010). Similarly, H101 has been combined with chemoradiotherapy in patients with stage IIB or III cervical cancer (Zhang et al., 2023). Collectively, these studies have reported encouraging results regarding treatment tolerability and antitumor activity. Collectively, these studies have reported encouraging results regarding treatment tolerability and antitumor activity (DeWeese et al., 2025). In addition, adenovirus-based OVs combined with intensity-modulated radiation therapy have demonstrated promising outcomes in phase I and II trials, including a reduction in positive biopsy rates among patients with high- or intermediate-risk disease (Freytag et al., 2007a; Freytag et al., 2014).

Overall, the promising results of combination therapies involving OVs highlight a compelling synergistic strategy. By integrating the complementary mechanisms of different treatment modalities, these approaches aim to overcome tumor immune evasion and resistance pathways, ultimately improving therapeutic efficacy and patient outcomes.

### 5.2 Understanding the results of clinical trials of genitourinary tumors

While the use of OVs has shown promise in the treatment of BC, outcomes in PC have been comparatively less encouraging. Although positive markers have been reported, several trials have demonstrated more modest improvements relative to BC. Among the factors underlying OVs’ underperformance in PC is the inherently immunosuppressive microenvironment of PC relative to BC, particularly given that a central mechanism of OV activity involves inducing ICD and apoptosis. The immunologically “cold” tumor phenotype is characterized by low tumor mutational burden and reduced neoantigen expression, limiting immune detectability. This is further compounded by impaired antigen presentation, including downregulation of MHC-I, as well as by the production of immunosuppressive cytokines and chemokines that collectively inhibit effective immune responses (Novysedlak et al., 2024).

Additional factors may also be related to OV delivery strategies. For instance, a lack of synergistic effects between OV therapy and docetaxel in PC has been reported following intravenous administration of a reovirus, potentially resulting in reduced viral replication within the TME (Eigl et al., 2018). In contrast, intraprostatic administration of the same formulation has been associated with increased cytotoxic lymphocyte infiltration and activation of apoptotic pathways (Thirukkumaran et al., 2010). The viral backbone itself may also contribute to these differences, as adenovirus-based formulations have shown more promising results compared to reovirus platforms (DeWeese et al., 2025; Freytag et al., 2007a; Freytag et al., 2007b). Furthermore, due to the anatomical localization of the prostate, access to the tumor is inherently restricted, making therapeutic administration more challenging, particularly when compared to the relatively straightforward intravesical route commonly used in BC (Xia et al., 2025).

Collectively, these factors suggest that the clinical potential of OVs in GU cancers may be constrained by host immune competence, delivery strategies, and the viral backbone itself. These variables should be carefully considered in the design of novel preclinical studies and cancer models.

## 6 Future perspectives

With the emergence of new possibilities for cancer treatment, including the promising use of OVs, there is also a need for extremely specific therapies that do not affect, or have only a very low chance of affecting, healthy cells, and the next wave of OV design is rapidly fulfilling this role. OVs are versatile in their mechanism of action, lysing tumoral cells while simultaneously stimulating the immune system and serving as vectors for gene therapy (Zhang and Rabkin, 2021; Zhou et al., 2025; Kaufman et al., 2015; Rahman and McFadden, 2021).

Viruses are now being conceived as multifunctional immune-activating platforms (Kennedy et al., 2020; Nguyen et al., 2022; Wang L. et al., 2022). However, payload engineering is equally important; cytokines (such as IL-12), GM-CSF, and TNF-related ligands have been tested, but in the future, it will be very important to focus on modular payloads, in which combinations of stimulatory molecules can be tailored to specific patient immunological deficits (Kaufman et al., 2022; Yin and Wang, 2024; Zheng et al., 2025; Martinez-Quintanilla et al., 2019).

CRISPR/Cas9 is a tool that enables the optimization of cancer immunotherapy through genetic manipulation. Early-stage research is currently exploring CRISPR-enabled editing to generate “smart OVs” that recognize and replicate preferentially in tumors defined by molecular signatures (Wang L. et al., 2022). The oncotropism of OVs is generally multifactorial, depending not only on the virus itself but also on tumor-specific characteristics, such as receptors, signaling pathways, tumor accessibility, metabolic alterations, and immune system escape, among many other factors (Yin and Wang, 2024; Harrington et al., 2019; Rahman and McFadden, 2021).

In this context, biomarker-driven patient selection is likely to become increasingly relevant for the clinical translation of OVs. Predictive biomarkers of response may include tumor expression of viral entry receptors, defects in intrinsic antiviral pathways such as type I interferon signaling, permissiveness to viral replication, and baseline immune contexture. Immune signatures such as pre-existing CD8^+^ T Cell infiltration, dendritic cell abundance, interferon-stimulated gene expression, and the balance between inflamed and immune-excluded phenotypes may help identify tumors more likely to benefit from OV-induced immune remodeling. In parallel, viral tropism determinants, including receptor availability, intracellular antiviral restriction mechanisms, and tumor-specific transcriptional or metabolic states, may directly influence infection efficiency and oncolysis. Together, these parameters may support a more rational matching of OV platforms to individual patients, moving the field toward biomarker-guided precision viro-immunotherapy (Harrington et al., 2019; An et al., 2018; Inoue et al., 2025).

## 7 Discussion

Tumors exhibit great heterogeneity, and this is one of the main reasons for the need for diversified treatment and a multi-pronged approach to achieve more effective treatment. In this context, oncolytic virus therapy shows great promise (Bi S. et al., 2025).

Oncolytic virotherapy has progressed from an experimental concept to a clinically validated modality, with BC leading the way through the FDA approval of Nadofaragene Firadenovec for BCG-unresponsive tumors, a landmark (Steinmetz et al., 2024; Boorjian et al., 2021; Moyer et al., 2026), but late-phase success in PC or RCC remains elusive (Kokorovic et al., 2021). In PC, biomarkers such as PSA, PSMA (prostate-specific membrane antigen), and androgen receptor signaling profiles may guide not only disease monitoring but also vector design. Beyond disease-specific markers, broader determinants of OV response, such as antiviral signaling defects, immune infiltration patterns, and viral receptor expression, should also be considered in future biomarker frameworks. For instance, promoters responsive to PSMA have already been used to restrict viral replication to prostate tissue, reducing the risk of off-target toxicity (Lee et al., 2004; Yang et al., 2022).

In GU cancers, early results with CAN-2409, Pexa-Vec, and VSV-based constructs highlight potential but also underscore persistent challenges in delivery, immune evasion, and scalability (Ahmed et al., 2024; Herman et al., 1999; Breitbach et al., 2015). Across all three malignancies (prostate, bladder, and kidney), the most consistent lesson is that OVs are not “standalone weapons”; their true therapeutic value emerges when they are combined with ICIs, radiotherapy, or targeted drugs, exploiting viral priming to convert immunologically “cold” tumors into “hot” ones (Kong et al., 2025; Gujar et al., 2018). Phase III trials for CG0070 in BC and advanced trials of CAN-2409 in PC will be pivotal for OV development. If positive outcomes are achieved, these studies may validate intratumoral and locoregional administration strategies as clinically sustainable approaches. While proof of concept has been established in multiple clinical trials, the next decade will determine whether OVs can attain regulatory maturity in GU oncology (Wang L. et al., 2022; Fretwell and Houldsworth, 2025).

Clinical trial models are constantly evolving “basket trials”, where OVs are tested across multiple tumor types defined by shared molecular features, and are emerging as an efficient way to accelerate clinical translation. This model is particularly relevant for rare subsets of GU cancers where patient accrual is traditionally difficult (Wang L. et al., 2022). At the same time, critical challenges remain, since antiviral immunity limits repeat dosing, and tumor selectivity must be tightened to avoid toxicity. Meanwhile, good manufacturing practice-grade manufacturing at sufficient scale remains an obstacle to broad adoption. However, innovation is meeting these challenges head-on. Advances in tumor-specific promoters, encapsulation strategies, and CRISPR-driven engineering are progressively reshaping safety and efficacy profiles (Wang L. et al., 2022; Xia et al., 2025; Wu et al., 2025).

Ultimately, the trajectory of OVs in GU oncology is one of cautious optimism. The field has moved beyond proof-of-principle into a phase of refinement and real-world validation. If the ongoing late-phase trials report durable clinical benefits, OVs may soon join ICIs and targeted agents as mainstream components of GU cancer management. More than a new drug class, OVs embody a conceptual shift, using viruses not as enemies, but as programmable allies in the fight against cancer. Taken together, the future of OVs in GU cancers is likely to reflect an intersection of precision medicine, advanced bioengineering, and clinical trial innovation, transforming OVs from niche therapeutics into a standard-of-care pillar (Zhang and Rabkin, 2021; Zhou et al., 2025).

**FIGURE 2:**
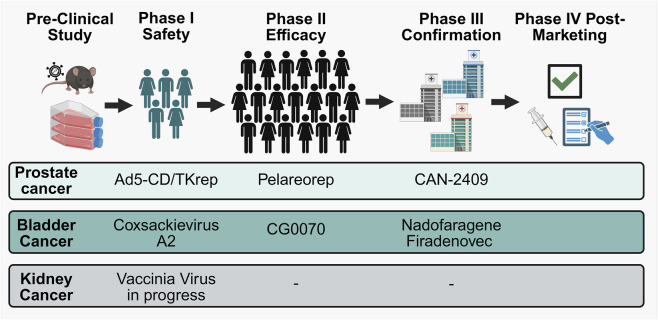
Clinical development landscape of oncolytic virus–based treatments in genitourinary malignancies. Clinical trials evaluating oncolytic virus-based therapies are currently underway in genitourinary cancer malignancies, with most studies focusing on bladder cancer and prostate cancer. These therapeutic strategies aim to enhance antitumor activity and improve clinical outcomes. In contrast, a limited number of clinical studies have been conducted to date in renal malignancies.

## Glossary

ATP: Adenosine triphosphate
BC: bladder cancer
BCG: *Bacillus* Calmette–Guérin
CAN-2409: aglatimagene besadenovec
ccRCC: Clear cell renal cell carcinoma
CG0070: Cretostimogene Grenadenorepvec
CR: Complete response
CRPC: Castration-resistant prostate cancer
DAMPs: Damage-associated molecular patterns
GM-CSF: Granulocyte-macrophage colony stimulating factor
GU: Genitourinary
HSV: Herpes Simplex Viruses
HSV-1: Herpes simplex virus type 1
HSV-TK: Herpes simplex virus thymidine kinase gene
ICD: Immunogenic cell death
ICI: Immune checkpoint inhibitor
MHC-I: Major histocompatibility complex Class I
MIBC: Muscle-invasive bladder cancer
MV: Measles virus
NMIBC: Non-muscle-invasive bladder cancer
OV: Oncolytic virus
PAMPs: Pathogen-associated molecular patterns
PC: Prostate cancer
PD-1: Programmed Cell Death Protein 1
PD-L1: Programmed Cell Death Ligand 1
PSA: Prostate-specific antigen
PXVs: Poxviruses
RCC: Renal cell carcinoma
RP1: Vusolimogene oderparepvec
tk: Thymidine kinase
TME: Tumor microenvironment
T-VEC: Talimogene Laherparepvec
VACV: Vaccinia virus
VEGF: Vascular endothelial growth
VEGF-TKIs: Vascular endothelial growth factor tyrosine kinase inhibitors
*VHL*: Von Hippel-Lindau gene
VSV: Vesicular stomatitis virus
